# Sanitation and Infectious Diseases

**Published:** 1884-01

**Authors:** 


Sanitation and Infectious Diseases.—Whether infectious diseases always originate from organisms or other products of animals already diseased, or from innocuous germs which afterwards develope infection, or whether *de novo* origin is possible, is, just at present, a much debated question.

Professor Williams in his address at the opening of the New Veterinary College, Edinburgh, expresses views favorable to the spontaneous origin of such diseases, and says: "If this opinion were to prevail," referring to origin by infection only, "what benefits are we to derive from sanitation, purity of atmosphere, good drainage, and conditions which, in this country at least, have almost abolished that dire and fatal malady, glanders, a disease which was rife in the memory of many of us. Glanders is now said never to originate spontaneously, and I believe that, under certain conditions, there is a deal of truth in the statement. If we compare the condition of our stables and the general treatment of horses with those of, say, twenty years ago, we can see some force in the conclusion, but if the surroundings of horses were now as they were in the past, I have no hesitation in stating that there would be numerous instances of glanders of spontaneous origin. Having, then, almost abolished glanders by improved sanitation, is it not reasonable to suppose that we may yet trace the conditions giving origin to all contagious diseases, and having found these out, be able to abolish them entirely?"

On the other hand we find Professor Walley giving expression in the course of his introductory lecture delivered at the Edinburgh Veterinary College, to the following opinions:

"While the doctrine of abrogenesis may be considered as annihilated, there is still room for differences of opinion as to the probability or possibility of comparatively harmless organisms, contracting in some way or other virulent and destructive powers. On the whole, perhaps, this modified doctrine commends itself more to the minds of scientific men than does the more radical one of spontaneous generation. Allowing that any particular disease is due to a recognized micro-organism, we cannot for a moment suppose that such an organism can spring out of nothing, or out of inert matter. I suspect Darwin, when he enunciated his theory of evolution of the higher organisms, little thought it would be one day applied to the infinitely lower; yet such is the case, and, perhaps, it is the best way of reconciling the believer in spontaneous generation to the test of his favorite bantling.

"On a former occasion I said that the question may yet be asked, is it not possible for that which, under ordinary and natural circumstances, is harmless or inert, to acquire specific characters when placed under extraordinary and abnormal conditions?

"Though a living unit may not actually spring out of dead matter, may not pre-existing units posssessed of definite qualities become so altered in their nature as, in their results at least, to present a striking contrast to their progenitors?

"Organisms, I think, it is now generally admitted, are pretty much harmless, providing the one important factor, a suitable soil, is not forthcoming, just as the seed will not grow if the soil in which it is sown possesses not the necessary elements for its nutrition, as the body cannot live if the food presented to it is deficient in the requisite constituents for its growth and nourishment; so will organisms be rendered inert, or perish, if the ground on which they are sown is not prepared for them."

Dr. W. B. Carpenter, in an address before the British Association for the Advancement of Science, states that "the contagia of the several infectious diseases are due to the progressive development, in hitherto innocent germs, of the conditions by which they acquire their infective activity and that the development of a mild or a potent infection was, in the main, a matter of cultivation."

While we incline to reject the spontaneous origin theory of infectious diseases, we leave the reader to solve the question as he pleases and turn to a more practical one, namely: Is sanitation effective against contagion?

According to Professor Williams we are to look for no other result in adopting sanitary measures than the abolishment of those conditions which favor the tendency to the spontaneous development of infectious diseases.

If we take then the other view of the origin of these diseases, namely, that by infection, are we to conclude, for that reason, that no benefits are to be derived from adopting measures of prophylaxis? Assuredly not. Far otherwise. The very fact that the development of infectious diseases depends on the character of the soil on which the germ locates itself and on the pathogenetic condition of that germ demonstrates the necessity of enforcing strict hygienic rules, both personal and public, for we know that infection carrying microbes thrive in unclean, badly drained and badly ventilated localities, and die when opposite conditions prevail, and that harmless germs continue harmless in sanitary surroundings.

The state of the general health of animals exposed to infection will decide whether they escape attack or not, hence care should be taken of their food and to maintain their hygienic condition up to the highest standard during epidemics.

Quarantine regulations would be entirely useless if the spontaneous origin theory were held, and who can doubt their value in checking the spread of glanders, anthrax, pleuro-pneumonia, etc.? C.

